# Antagonistic action on NMDA/GluN2B mediated currents of two peptides that were conantokin-G structure-based designed

**DOI:** 10.1186/s12868-017-0361-4

**Published:** 2017-05-16

**Authors:** Edwin A. Reyes-Guzman, Nohora Vega-Castro, Edgar A. Reyes-Montaño, Esperanza Recio-Pinto

**Affiliations:** 1 Department of Anesthesiology, Perioperative Care and Pain Medicine, NYU Langone Medical Center, 180 Varick Street, Room 677, New York, NY 10014 USA; 2 Grupo de Investigación en Proteínas, Departamento de Química, Universidad Nacional de Colombia, Cra 30 No 45-03 Edificio 451 Lab 201-1, Bogotá, Colombia

**Keywords:** NMDA, Conantokin-G, Hippocampal neurons, GluN2B, GluN2A

## Abstract

**Background:**

The GluN2B subunit of the *N*-methyl-d-aspartate receptor (NMDAr) modulates many physiological processes including learning, memory, and pain. Excessive increase in NMDAr/GluN2B activity has been associated with various disorders such neuropathic pain and neuronal death following hypoxia. Thus there is an interest in identifying NMDAr antagonists that interact specifically with the GluN2B subunit. Recently based on structural analysis between the GluN2B subunit and conantokin-G, a toxin that interacts selectively with the GluN2B subunit, we designed various peptides that are predicted to act as NMDAr antagonists by interacting with the GluN2B subunit. In this study we tested this prediction for two of these peptides EAR16 and EAR18.

**Results:**

The effects of EAR16 and EAR18 in NMDA-evoked currents were measured in cultured rat embryonic hippocampal neurons and in HEK-293 cells expressing recombinant NMDAr comprised of GluN1a–GluN2A or GluN1a–GluN2B subunits. In hippocampal neurons, EAR16 and EAR18 reduced the NMDA-evoked calcium currents in a dose-dependent and reversible manner with comparable IC50 (half maximal inhibitory concentration) values of 241 and 176 µM, respectively. At 500 µM, EAR16 blocked more strongly the NMDA-evoked currents mediated by the GluN1a–GluN2B (84%) than those mediated by the GluN1a–GluN2A (50%) subunits. At 500 µM, EAR18 blocked to a similar extent the NMDA-evoked currents mediated by the GluN1a–GluN2B (62%) and the GluN1a–GluN2A (55%) subunits.

**Conclusions:**

The newly designed EAR16 and EAR18 peptides were shown to block in reversible manner NMDA-evoked currents, and EAR16 showed a stronger selectivity for GluN2B than for GluN2A.

## Background

*N*-methyl-d-aspartate receptors (NMDAr) are inotropic glutamate receptors (iGluRs) that contribute to multiple neuronal functions such as [1–3]. However, excessive NMDAr activity can lead to neuronal dysfunction and neuronal death [4, 5]. Functional NMDAr require two GluN1 and two GluN2 subunits [4, 6–8]. The GluN2 subunit can be replaced by a GluN3 subunit, but when present the GluN3 subunit decreases the NMDAR activity [9, 10]. There are four GluN2 subunits (GluN2A, B, C and D) [11]. An increase in the expression of GluN1, GluN2A, and GluN2B subunits in the hippocampus contribute to neurological problems that develop following traumatic brain injury [12]. Increase in the activity/expression of GluN2B has been postulated to contribute to neuronal damage following stroke, as well as to the development of diseases such as Parkinson, Huntington, Alzheimer and chronic neuropathic pain [11].

NMDAr are similar to other iGluRs in that each subunit has four domains, which include the amino-terminal domain (ATD), an agonist/ligand binding domain (ABD), a trans-membrane domain (TMD) and a carboxyl terminal domain (CTD) [13–15]. In GluN2 subunits, the ATD domain contains binding sites for allosteric inhibitors such as zinc [16] and ifenprodil [17]. The ABD domain in GluN1 and GluN3 binds the coagonist glycine, whereas the ABD domain in GluN2 binds the agonist glutamate [11, 13–15]. Some peptides isolated from marine cone snails have shown specificity for GluN2 subunits of the NMDAr [18]. One member of the conantokin family, conantokin-G is a 17-residue peptide that shows selectivity for NMDAr containing the GluN2B subunit [18]. Although, conantokin-G shows high selectivity for GluN2B, its reversibility appears to be very slow and incomplete on neuronal cells [19]. We designed a number of peptides with potential GluN2B–NMDAr antagonistic activity, this was done by using point mutations on the conantokin-G sequence and evaluating their structure as well as their binding capacity to the ligand binding domain of the GluN2B subunit by using various in silico evaluations. Here we tested this prediction for two of these peptides, EAR16 and EAR18, and found that both of these peptides blocked in a highly reversible manner NMDA-evoked currents in hippocampal neurons, and that EAR16 was more selective for NMDArs containing the GluN2B than for those containing the GluN2A subunit. The high reversibility of both of these peptides and the higher selectivity of EAR16 for the NMDArs containing the GluN2B increase their potential as pharmacological agents.

## Methods

### Peptides

The peptides EAR16 (GEDD**L**QD**N**QDLIRDKSN) and EAR18 (GEDD**Y**QD**A**QDLIRDKSN) were designed in the Bioinfo Grip laboratory at the National University of Colombia (patent in progress, the peptides are commercially available) to interact with the ligand binding domain (LBD) of the GluN2B subunit. The peptide design and their in silico evaluation was achieved by using point mutations on the conantokin-G sequence and evaluating their binding capacity with AutoDock Vina [20] and Rosetta FlexPepDock server [21], by using a structural model of GluN2B that was developed in our laboratory (Reyes-Guzman et al. in press), as briefly described below. As a control, we initially selected a scramble peptide with the same length (17 a.a.) like is usual in this kind of design. However, this scramble peptide displayed agonist-like activity, potentially by interacting at a site different than the LBD. Hence we selected another non-related peptide (EAR7) as control (TKRSSRAFRE), one that was designed to interact with an intracellular site, the C-terminal domain (CTD) of GluN2B subunit [22]. EAR16 and EAR 18 show good water solubility (http://pepcalc.com) and have similar hydrophobicity levels: 19.36 and 19.65, respectively (https://www.thermofisher.com/co/en/home/life-science/protein-biology/peptides-proteins/custom-peptide-synthesis-services/peptide-analyzing-tool.html). Permeability was predicted using CellPPD (http://crdd.osdd.net/raghava/cellppd/index.html) which provided negative CPP values for these peptides (−0.06, −0.57 and −0.57 for EAR7, EAR16 and EAR18, respectively), indicating that they are non-cell permeant (CPP less than 0, indicate that the peptide is non-cell permeant). The peptide synthesis was made by a commercial supplier (Peptide 2.0 in Sterling, VA. USA). These peptides show no modifications at either terminal, their N terminal is a free amine and the C terminal is a free acid (analysis performed by Peptide 2.0. The peptide purity and other properties are shown in Table 1.

**Table 1:** Peptide Properties

	Purity^a^ (%)	MW (g/mol)^b^	MS (M + H^+^)^c^	MS (M + Na^+^)^d^
EAR16	99.28	1975.03	1976.00	1997.10
EAR18	98.22	1982.02	1983.17	2004.84
EAR7	98.87	1237.39	1237.71	–

These values were provided by the company (Peptide 2.0 in Sterling, VA. USA)

^a^Purity determine by high-performance liquid chromatograph (HPLC) (HPLC 220 nm, C18, linear gradient)

^b^MW: molecular weight, theoretical

^c^MS (M + H^+^): hydrogen cation mass spectrometry analysis

^d^MS (M + Na^+^): sodium ion mass spectrometry analysis

### Peptide three-dimensional structure

For each peptide a three-dimensional structure model was generated using UCSF (University of California San Francisco) Chimera software (http://www.cgl.ucsf.edu/chimera/, [23]), and a refined three-dimensional structure was obtained in an hydrophilic environment using PEPstr (Peptide Tertiary Structure Prediction Server; http://www.imtech.res.in/raghava/pepstr/) as previously described [22].

### Structural model of the GluN2B

Details of this model are described in a separate publication [22]. Briefly, this model was obtained by using the amino acids 1-817 of the reported sequence of the GluN2B subunit (Ensembl ENSRNOP00000011697, *Rattus norvegicus*). This amino acid sequence includes the amino terminal (ATD) and the ligand binding (LBD) domains. The GluN2B structural model was predicted using I-TASSER (http://zhanglab.ccmb.med.umich.edu/, [24]), this software used several templates to determine the best model. The best model (based on the C-Score and TM-score) was obtained when the template used was the inotropic glutamate AMPA (α-amino-3-hydroxy-5-methyl-4-isoxazolepropionic acid) receptor (PDB 3KG2) [8]. This template has been previously used to elucidate other functions of glutamate receptors [25]. The C-score usually ranges between −5 and 2, the most positive the value the better the model topology, the GluN2B model selected has a C-core of 0.01 [22]. The TM-score provides a value of the structural correspondence between two structures (GluN2B-template), values higher than 0.5 indicate that the model topology is likely to be correct [24], the selected GluN2B model has a TM-score of 0.71.

The selected GluN2B model (using I-TASSER) was further evaluated using PDBsum (http://www.ebi.ac.uk/pdbsum/, [26]), that provides various parameters including hydrophobicity profile, solvent accessibility, prediction of ligand binding sites. The secondary structure of the model was also evaluated using Ramachandran plots (Procheck in PDBsum), which provides the percentage of structural regions that are either “favorable”, “allowed” or “not-allowed”. A model with good stereo-chemistry should have close to 90% of the residues in structurally favorable regions [27]. The selected model has 86.7% of the residues located in structurally “favorable” regions, and 10.9% of the residues in “allowed” regions [22]. The model has structural topology comparable to that described for other inotropic glutamate receptors [25], including the ATD, LDB and the transmembrane (TDM) domains. The ATD and LBD structural configurations are composed of mostly α helices and allows the binding of modulators an agonists [22].

### Molecular docking

Molecular docking was performed with the obtained three-dimensional structures of the peptides and of the GluN2B subunit by using “Autodock Vina” (http://vina.scripps.edu/, [20]). A Grid Box was generated at the center of the LBD of the GluN2B model to which the corresponding hydrogen atoms and chargers were assigned. The Grid Box was 1 Å, in the software “completeness” was set to 10 and the number of results per Docking was set to 10, for each peptide. The peptides corresponded to the ligands. The best conformations and orientations, which displayed the lowest values for binding energy (−6.6 and −7.7 kcal/mol for EAR16 and EAR18, respectively), were visualized using Autodock Tools (v. 1.5.4.).

The Docking results were then analyzed with the Rosetta FlexPepDock web server (http://flexpepdock.furmanlab.cs.huji.ac.il/index.php, [21]), which refines the peptide-receptor interaction by optimizing the peptide orientation within the binding site using the Monte-Carlo method and focusing on energy minimization of the ligand with the receptor. With the starting structure FlexPepDock realized 200 independent simulations, 100 simulations were done under high resolution. The other 100 simulation included a step of pre-optimization at low resolution, followed by a method of refinement of high resolution. This resulted in 200 models for each peptide, we selected the best one for each peptide based on the score provided by the punctuation function of Rosetta (675.461 and 755.051 for EAR16 and EAR18, respectively) [28]. The docking images were generated using UCSF Chimera (http://www.cgl.ucsf.edu/chimera/, [23]).Interactions between peptide-receptor were determine using Discovery Studio Visualizer v4.1 software (http://accelrys.com/products/discovery-studio/visualization.html).

### Hippocampal cultures

Pregnant rats were maintained and used following the guidelines approved by the New York University Langone Medical Center Institutional Animal Care and Use Committee. Pregnant rats were killed using CO_2_ followed by cervical dislocation. Day 18 embryos (E18) were removed by caesarian section. Dissociated hippocampal cultures were prepared as previously described [29, 30]. Cells were plated on 12 mm coverslips (7.0 × 10^4^ cells/well, 24 well plates) with neurobasal medium supplemented with B27 (Gibco Invitrogen, ThermoFisher Scientific, USA) or with 2% bovine fetal serum and used between 7 and 37 days in vitro; the data was collected from 57 cells that had on average 14.9 days in vitro (14.9 ± 10.8 mean ± SD). Coverslips were pre-treated by placing 50 µL of poly-d-Lysine (37.5 μg/mL, Sigma-Aldrich, USA) and Laminin (2.5 μg/mL, Invitrogen) in the middle of the coverslip for 5 min at room temperature (RT).

### Transfection of HEK-293 cells

The plasmids pEGFP-NR1a (GluN1a) (Addgene plasmid # 17926), pEGFP-NR2B (GluN2B) (Addgene plasmid # 17925), and pEGFP-NR2A (GluN2A) (Addgene plasmid # 17924) were a gift from Stefano Vicini [31]. HEK-293 cells (ATCC, Manassas,Virginia, USA) were grown on 12-mm coverslips in 24 well plates. HEK-293 cells (60–70% confluent) were transfected using Lipofectamine 2000 according to the product instructions (Invitrogen), with a mixture of GluN1a and GluN2A subunits or a mixture of GluN1a and GluN2B subunits. The amount of cDNA for each plasmid was 0.25 µg/well. Cells were used 2–4 days following transfection. GFP signal was used to identify positively transfected cells.

### Electrophysiology

Electrophysiological recordings were performed under voltage-clamp by using the whole cell conformation [32, 33]. Intracellular solution (in mM): 110 Cs-gluconate, 20 CsCl, 10 Hepes, 10 EGTA, 4 Mg-ATP, 0.4 Na-GTP, pH 7.3 (adjusted with CsOH) [34]. Extracellular solution (in mM): 140 NaCl, 2.5 KCl, 2.0 CaCl_2_, 10 Hepes, 10 d-glucose, pH 7.4. Mg^2+^ was omitted to prevent the voltage-dependent block of the NMDAr ion currents [35, 36]. The following blockers were added to extracellular solution: 1.0 μM tetrodotoxin (TTX; Sigma, St. Louis, MO) to block Na^+^ channels [34]; 20 μM CNQX (6-cyano-7-nitroquinoxaline-2,3-dione, Sigma) to block AMPA and Kainate receptors, 50 μM Bicuculine (Sigma) and 100 μM Picrotoxin (Sigma) to block GABA receptors, and 1 μM Estricnin to block glycine channels. Currents were measured using borosilicate pipettes (1.5 mm outer diameter; 0.86 mm inner diameter with filaments, World Precision Instruments, Inc, Sarasota, FL) with resistances of 1–3 MΩ. Experiments were conducted at room temperature.

Currents were measured using a 2-electrode voltage clamp amplifier Axopatch 200B (Axon Instruments, Foster City, CA; Molecular Devices, LLC, Sunnyvale, CA). Output from the voltage clamp amplifier was sent to a microcomputer using a data acquisition interface (Digidata 1440A; Axon Instruments). Currents were filtered at 2.5 or 5 kHz (Axopatch 200B) and sampled at 10–50 kHz. For analysis the current traces were filtered at 1 kHz. Pclamp 10.4 (Axon Instruments) was used for data acquisition and data analyses.

**Fig. 1:**
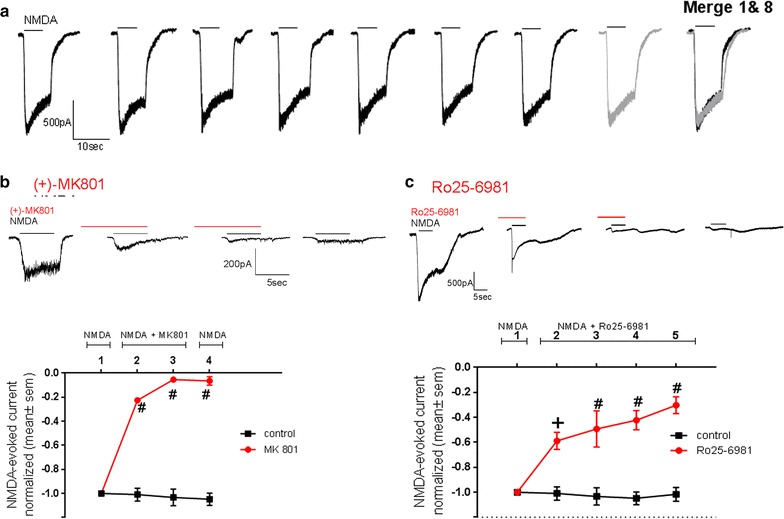
Evoked NMDA-currents in cultured hippocampal neurons are blocked by (+)-MK801 and Ro 25-6981. **a** Whole-cell NMDA-evoked inward currents (*opening downward*) induced by consecutive 5 s pulses of 100 µM NMDA + 10 µM Glycine, with a 4 min interval. The last image is an overlap of the first (*black*) and eighth (*gray*) NMDA-evoked current traces. **b** Inhibition of NMDA-evoked inward current by the general NMDAr antagonist (+)-MK801 (10 µM). *Top* shows traces of the NMDA-evoked inward currents and below the normalized area of the NMDA-evoked inward currents (−1.0 corresponding to the maximal value) for control (*black circles*); and before, during two consecutive exposures to (+)-MK801 and after removing (+)-MK801 (*red circles*). **c** Inhibition of NMDA-evoked inward current by the GluN2 specific blocker Ro 25-6981 (1 µM). *Top* shows the NMDA-evoked inward currents in a cell before, during two consecutive exposures to Ro 25-6981, and after removing Ro 25-6981. *Bottom* shows the normalized NMDA-evoked inward currents for control (*black circles*); and before and during four consecutive exposures to Ro 25-6981 (*red circles*). Significant different compared to the control group #p < 0.0001, +p < 0.001, two-way ANOVA with Sidak’s multiple comparison post-test; n (number of cells), control: n = 7; (+)-MK801: n = 3; and Ro 25-6981: n = 4 during the first three NMDA stimulations and 2 for the last two NMDA stimulations

Cells were held at −60 mV and the NMDAr-evoked currents were obtained by applying a 5 s pulse of NMDA (50 or 100 μM NMDA + glycine 10 μM) followed by a 10 s pulse of bath solution through a puffer (flow rate: 250 μL/min) located directly on top of the cells (perfusion manifold MMF; Scientifica, East Sussex, United Kingdom). Successive stimulations with NMDA were done every 4 min, which under control conditions resulted in NMDA-evoked Ca^2+^ currents of similar magnitude over time (Fig. 1a). When the effects of (+)MK-801(Dizocilpine, Tocris) a general NMDAr blocker, Ro 25-6981 (Sigma) a specific GluN2B/NMDAR blocker), and peptides (EAR16 and EAR18) were evaluated, the cells were first exposed to two or three consecutive NMDA pulses (4 min interval) to assure that the cell recording was stable, then the blocker (or peptide) was applied 10 s prior and during the following NMDA pulse. In all cases the NMDA pulses were followed by a 10 s pulse of bath solution through the puffer. The chamber was continuously perfused with the bath solution (flow rate: 400 μL/min), except when the puffer was on. Peptides were added at a final concentration of 10, 100 and 500 μM. The area of the NMDA-evoked inward currents was measured over a period of 5 s, from the onset of the NMDA-evoked inward current. The areas were normalized using the NMDA-evoked inward current measured prior to the addition of the blocker or peptide (−1: maximal inward current; 0: no current). Figures were done using GraphPad Prism v6. Results are expressed as mean and standard error of mean (mean ± S.E.M).

### Statistics

When comparing two groups with various stimulations we used Two-way ANOVA (analysis of variance) followed by Sidak’s multiple comparison post-test. When comparing more than two groups with various stimulations we used Two-way ANOVA followed by Dunnett’s multiple comparison post-test, as recommended by GraphPadPrism 7.02. Results are expressed as mean and standard error mean (mean ± S.E.M.).

## Results

### Electrophysiological evaluation of EAR16 and EAR18 peptides on hippocampal primary cultures

**Fig. 2:**
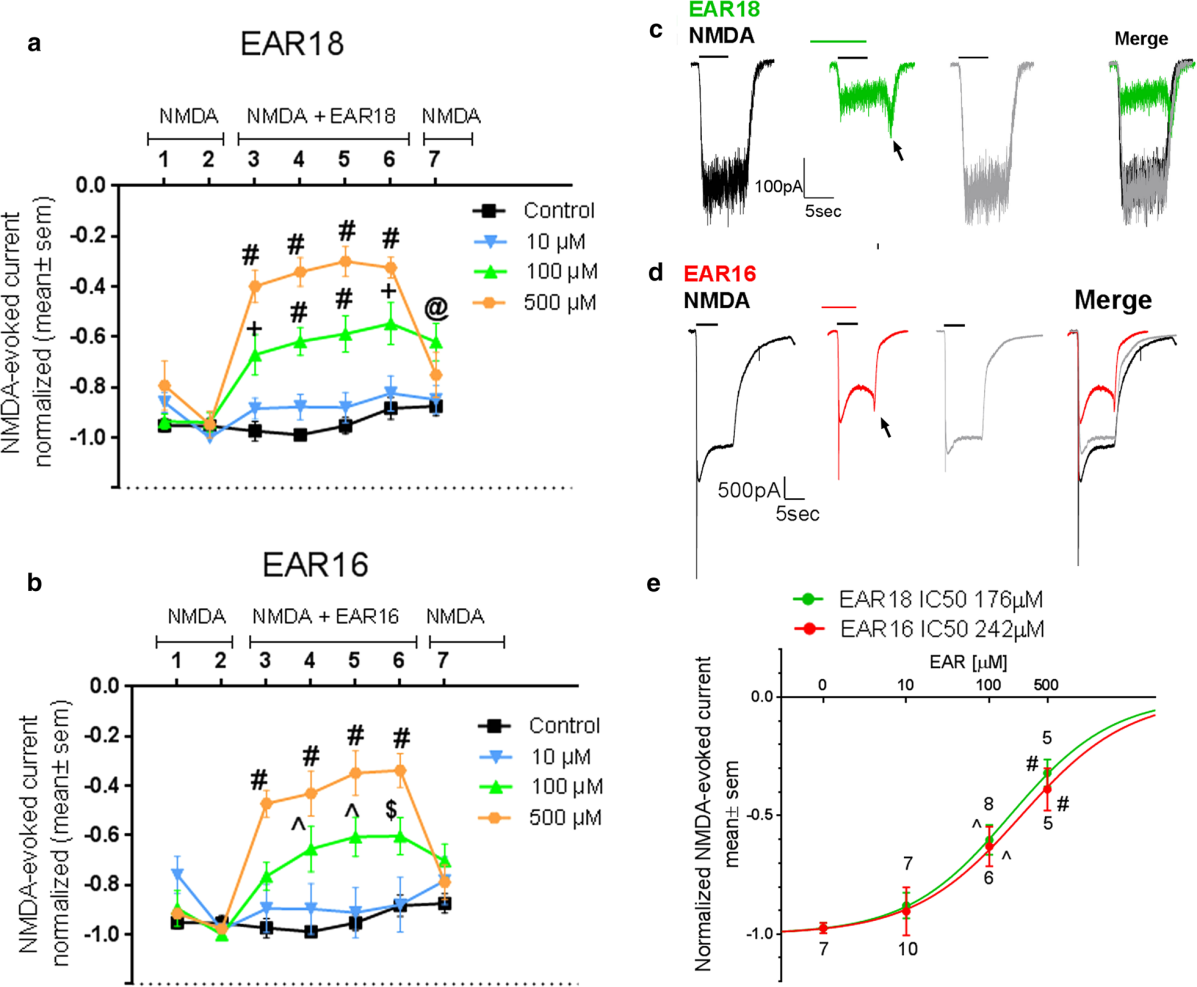
EAR16 and EAR18 peptides inhibit NMDA-evoked inward currents in hippocampal neurons. **a**, **b** The inhibitory effect on NMDA-evoked currents during consecutive exposures to either 10, 100 and 500 µM of EAR18 (**a**) and EAR16 (**b**). The areas of the NMDA-evoked inward currents were normalized to that measured prior to the addition of EAR18 (or EAR16) (−1: maximal inward current; 0: no current). Significant different compared to the control group. #p < 0.0001, +p<0.001, @p < 0.02, ^p < 0.01, $p < 0.05, two-way ANOVA with Dunnett’s multiple comparison post-test; n (number of cells) for each pulse were for Control (n = 7, 7, 7, 7, 6, 5, 4); for EAR18: 10 µM (n = 7), 100 µM (n = 8); 500 µM (n = 5); for EAR16: 10 µM (n = 10), 100 µM (n = 6, 7, 7, 6, 6, 6, 6, 5) and 500 µM (n = 7, 11, 11, 5, 5, 6, 5). **c**, **d** Show NMDA-evoked current traces before, in the presence of EAR16 (or ER18) and following washout. The *arrows* indicate that upon the simultaneous removal of NMDA + EAR, there is a transient increase in the NMDA-evoked inward current (see text for “Discussion” section). **e** Dose–response, the data was fitted to Y = Bottom + (Top − Bottom)/(1 + 10^((LogIC50-x)*HillSlope)). When the entire current was considered: *Bottom* = 0 no current, *top* = −1 (normalized maximal inward current), the IC50 s were 176 and 241 µM for EAR18 and EAR16, respectively

Primary hippocampal cultures were used between 4 and 20 days of culture, a period when the GluN2B subunit has been shown to be highly expressed [37, 38]. When NMDA stimulations were separated by a 4 min interval the magnitude of the NMDA-evoked Ca^2+^ currents was similar between consecutive stimulations (Fig. 1a), as reflected by the average behavior shown by the black squares in Figs. 1b, c and 2a, b. The general NMDA blockers (+)MK-801 (10 µM), blocked the NMDA-evoked currents (Fig. 1b). The magnitude of (+)MK-801-mediated block increased with exposures to the blocker and it was irreversible (Fig. 1b), as previously reported in hippocampal neurons [39]. In order to determine the contribution of the GluN2B to the NMDA-evoked response we exposed the hippocampal cells to a GluN2B selective blocker Ro 25-6981 (1 µM), at a concentration that is 100 fold higher that producing 50% inhibition of NMDA/GluN2B-mediated currents [40]. At this concentration Ro 25-6981 has minimal or no effects on voltage-dependent Na^+^ and Ca^+2^ channels [40]. Block with Ro 25-6981 showed no reversibility following 4 min of washout (Fig. 1c, top). We observed that Ro 25-6981 blocked 70% of the NMDA-evoked currents (Fig. 1c, bottom); hence under our experimental conditions about 70% of the NMDA-evoked currents in hippocampal neurons are mediated by activation of GluN2B/NMDAr. Moreover, the increase in the magnitude of the block observed following consecutive exposures to Ro 25-6981 is consistent with the blockers being use-dependent.

In hippocampal neurons, the effect of EAR16 and EAR18 on the NMDA-evoked currents was accessed by applying each peptide for four consecutive NMDA-evoked currents to determine whether these peptides blocked the NMDA-evoked current and if so whether their block displayed use-dependence behavior. EAR18 inhibited NMDA-evoked currents in a dose-dependent manner, and this inhibition was reversible (Fig. 2a, c). EAR16 had a similar inhibitory effect on NMDA-evoked currents in hippocampal neurons (Fig. 2b, d). For a given dose, the magnitude of the block mediated by EAR18 or EAR16 did not increase significantly with consecutive exposures (Fig. 2a, b); hence these peptides show no clear use-dependence (compared to that observed by Ro 25-6981 Fig. 1c). Examination of the current traces, show that following the simultaneous removal of the NMDA + EAR16 (or NMDA + EAR18) there was a relatively short-lived increase in the NMDA-evoked current, suggesting that the off rate of EAR16 (and EAR18) is fast enough that would allow binding of NMDA early on during washout (Fig. 2c, d indicated with arrows). Figure 2e shows the dose–response curves. When considering the total current magnitude the estimated IC50 values were similar for both peptides being 176 and 242 µM for EAR18 and EAR16, respectively (smooth lines). No inhibitory action was observed with the control peptide at 500 µM (data not shown).

### EAR16 displays higher selectivity than EAR18 for the GluN2B subunit over the GluN2A subunit

To test the selectively of these peptides for the GluN2B subunit, the blocking effect of these peptides was tested in HEK-293 cells expressing recombinant NMDAr comprised of either GluN1a–GluN2A or GluN1a–GluN2B subunits. The rationale for doing so is that these are the predominant GluN2 subunits in hippocampal neurons, moreover the amino acid sequence corresponding to the sequence used for the structural model of GluN2B (1-818) is conserved (71.9% identity) and of the LBD is highly conserved (95.5% identity) between GluN2A and GluN2B [25, 41, 42]. We selected 500 µM of each peptide to test their potency on HEK293 cells expressing recombinant NMDAr, since this peptide concentration produced a strong but not maximal block (~60%) on NMDA-evoked currents in hippocampal neurons (Fig. 2e). This should allow to determine whether or not these peptides displayed differences in potency between the GluN2B and GluN2A subunits.

**Fig. 3:**
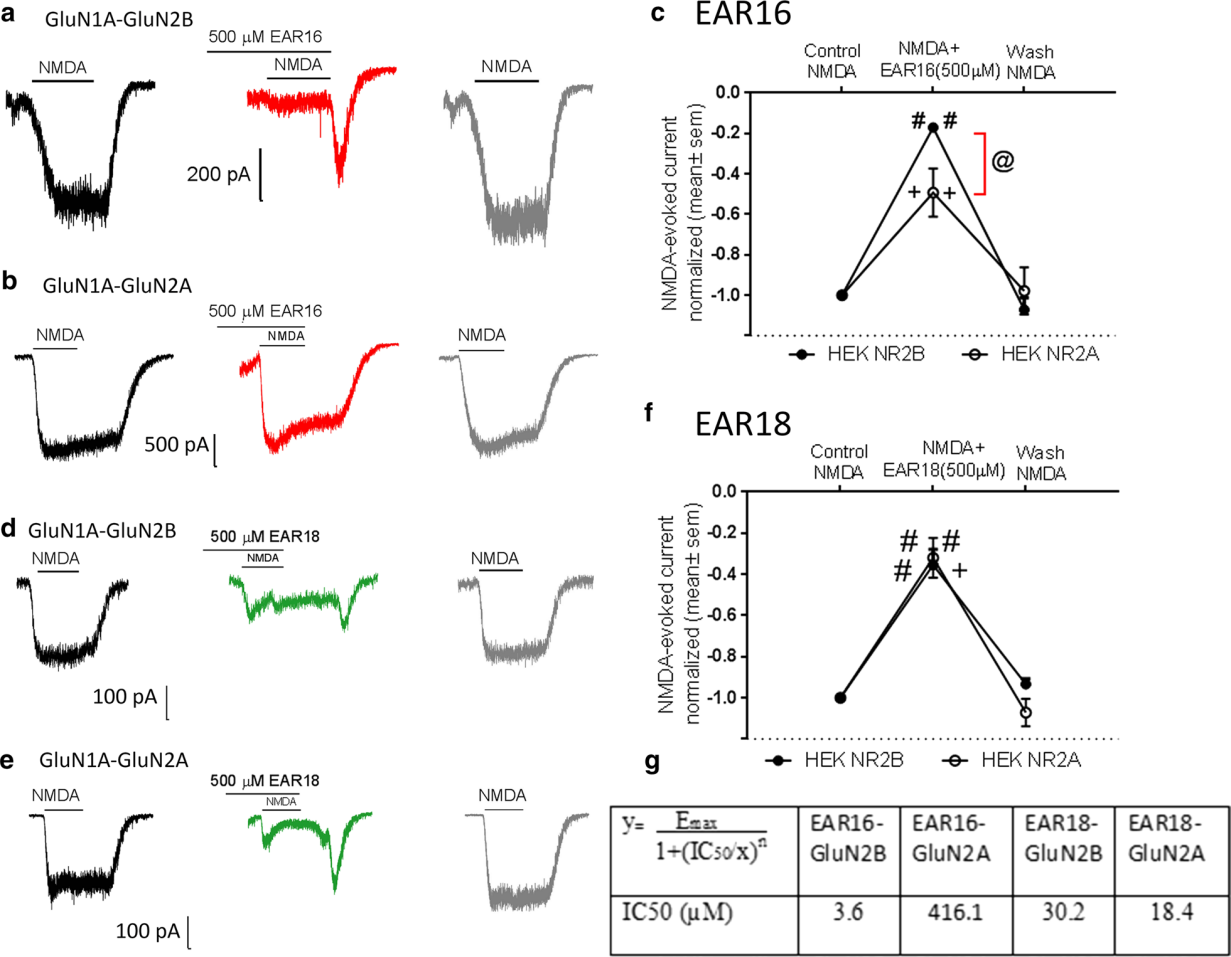
EAR16 but not EAR18 shows selectivity for GluN2B over GluN2A. **a**, **b** EAR16-mediated inhibition of NMDA-evoked inward currents on HEK-293 cells expressing GluN1A–GluN2B subunits (**a**) or expressing GluN1A–GluN2A subunits (**b**). **d**, **e** EAR18-mediated inhibition of NMDA-evoked inward currents on HEK-293 cells expressing GluN1A–GluN2B (**d**) or in cells GluN1A–GluN2A subunits (**e**). **c**, **f** The areas of the NMDA-evoked inward currents were normalized to that measured prior to the addition of EAR16 (**c**) or EAR18 (**f**) (−1: maximal inward current; 0: no current). No significant difference was found between “control-NMDA” and “wash-NMDA”. Significant different compared to “control-NMDA” (*left symbol*) or when compared to “wash-NMDA” (*right symbol*) #p < 0.0001, +p < 0.001, @p < 0.02, two-way ANOVA with Sidak’s multiple comparison post-test; n (number of cells) EAR16, n = 4 for GluN1A–GluN2B, and n = 4 for GluN1A–GluN2A; EAR18 n = 3 for GluN1–GluN2B (washout n = 2) and n = 4 for GluN1A–GluN2A

Figure 3 shows that as predicted EAR16 at 500 µM blocked more strongly NMDA-evoked currents in HEK cells transfected with the GluN1a–GluN2B subunits (Fig. 3a, e) than those in cells transfected with the GluN1a–GluN2A subunits (Fig. 3b, e). In contrast, EAR18 at 500 µM blocked to a similar level the NMDA-evoked currents in HEK-cells transfected with either GluN1a–GluN2B (Fig. 3c, f) or GluN1a–GluN2A subunits (Fig. 3d, f). The IC50 of each peptide for blocking GluN1a–GluN2B and GluN1a–GluN2A was estimated by using Eq. (1), where ‘x’ is the peptide concentration in log10 (500 µM in log10 = 2.69897); ‘y’ is the % inhibition at ‘x’ (from Fig. 3c, f); Emax: is the maximal inhibitory effect which we are assuming to be 100%; ‘n’ is the slope of the fitting, which it is assumed to be “1” since there is only a single recombinant NMDAr (containing either the GluN2B or the GluN2A).

1 $${\text{y}} = {\text{E}}_{\text{max} } /\left( {1 + {\text{IC}}_{50} /{\text{x}}} \right)^{\text{n}}$$

The results are shown in Fig. 3g, and indicate that EAR16 has about 100 fold higher affinity for GluN1a–GluN2B than for GluN1a–GluN2A (3.6 vs 416.1 µM). While EAR18 displayed a comparable affinity for both GluN1a–GluN2B and GluN1a–GluN2A (30.2 and 18.4 µM).

As observed in hippocampal neurons, in HEK-293 cells transfected with recombinant NMDAr the simultaneous removal of the NMDA + EAR16 (or NMDA + EAR18) also resulted in a short-lived increase in the NMDA-evoked current, suggesting that the off rate of EAR16 (and EAR18) was fast enough that would allow binding of NMDA early on during washout.

### Docking of EAR16 and EAR18 with the LBD of the GluN2B model

**Fig. 4:**
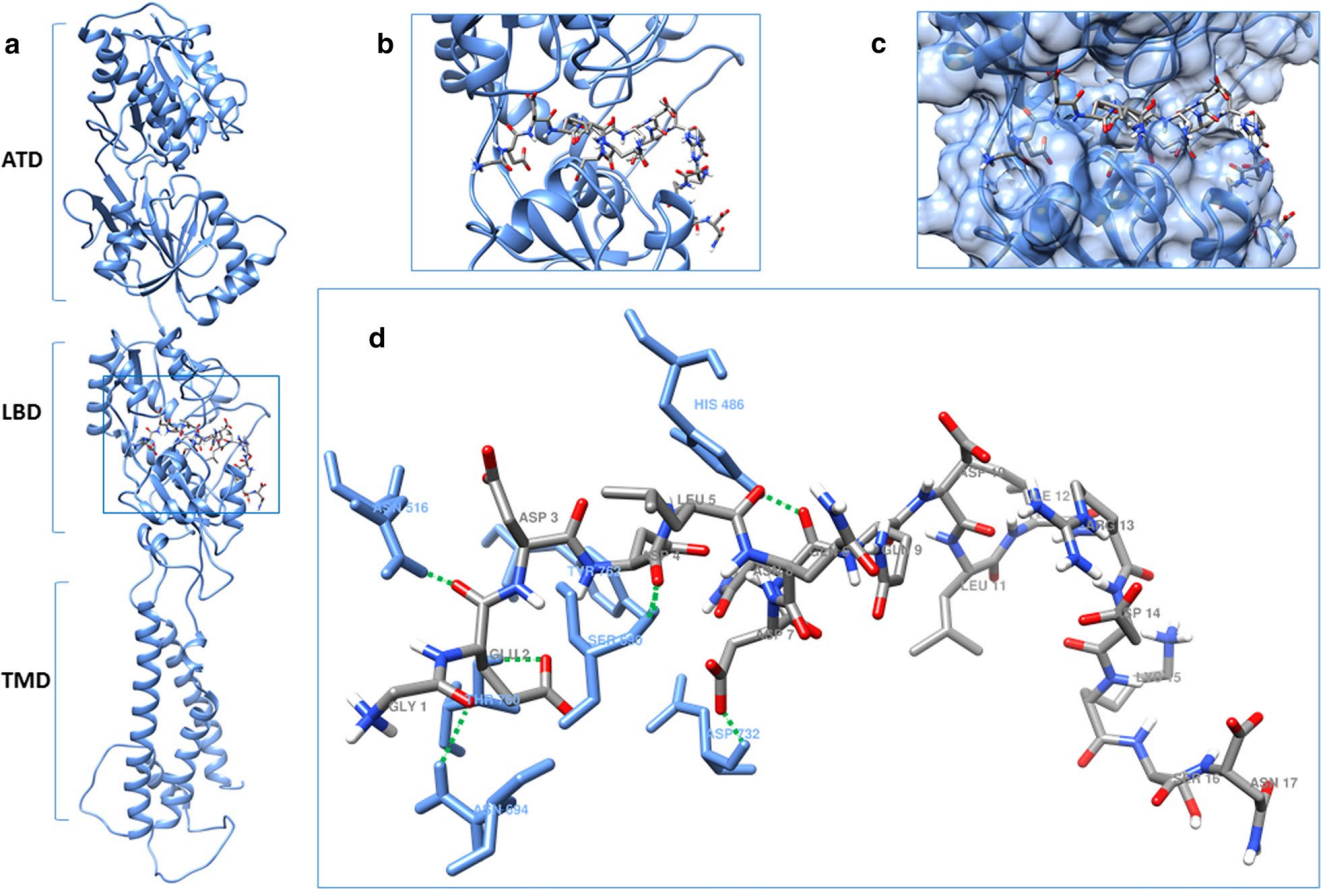
Schematic representation of docking of EAR16 with the LBD of GluN2B. **a**, **b** Docking of EAR16 with the LBD (ligand binding domain) in the GluN2B model. **c** Representation of the molecular surface of LBD in the GluN2B, and the peptide conformation. **d** Amino acid residues and labels of GluN2B (*light blue*) that form hydrogen bridges (*green dashed lines*) with EAR16. The amino acid residues for EAR16 are represented by element: *carbon gray*, nitrogen: *dark blue*, oxygen: *red* and, hydrogen: *white*; and the amino acid names in *gray*

Figure 4 shows the docking results of the interaction between the EAR16 and the GluN2B subunit. EAR16 binds to the GluN2B through 7 hydrogen bridges, 4 of them are with residues located at the LBD in GluN2B (His 486, Ser 690, Asp 732, Tyr 762) [43]. The EAR16 residues Asp 4 and 7 contribute to the formation of these hydrogen bridges. The residue Met 739, that has been reported to contribute to the molecular interaction between Con-G and GluN2B [44], is predicted to form a hydrophobic interaction (alkyl) with the Leu 11 of EAR16. The majority of the interactions involve the amino terminal of EAR16, similar to what has been described for conantokin-G [18], indicating that the substitution of the residue γ-carboxiglutamic acid (Gla) by Asp appears to emulate the Con-G interaction with the GluN2B.

**Fig. 5:**
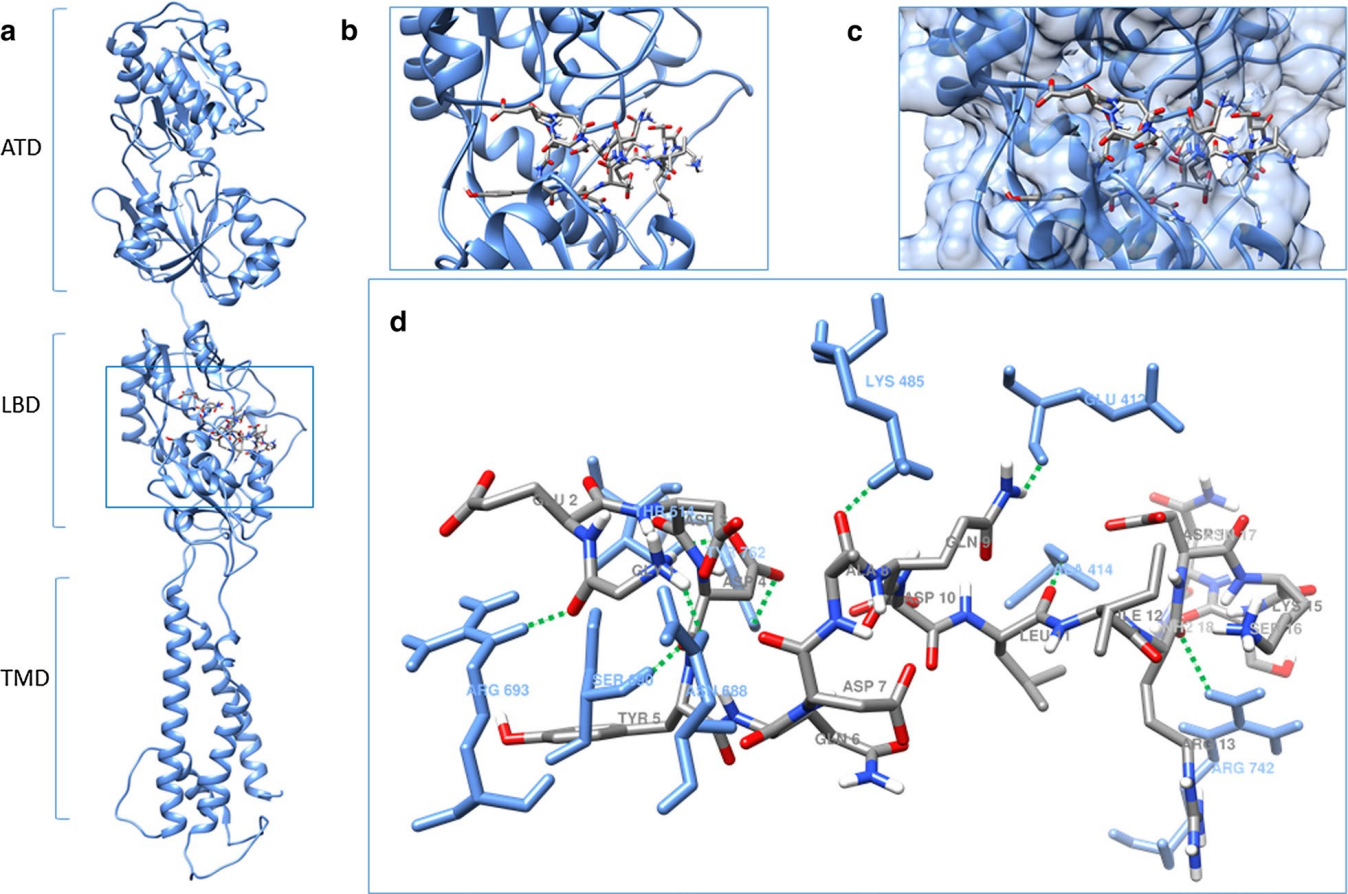
Schematic representation of docking of EAR18 with the LBD of GluN2B. **a**, **b** Docking of EAR18 with the LBD in the GluN2B model. **c** Representation of the molecular surface of LBD in the GluN2B, and the peptide conformation. **d** Amino acid residues and labels of GluN2B (*light blue*) that form hydrogen bridges (*green dashed lines*) with EAR18. The amino acid residues for EAR18 are represented by element: carbon: *gray*, nitrogen: *dark blue*, oxygen: *red*, and hydrogen: *white*; and the amino acid names are in *gray*

In the case of EAR18, the interactions involve 9 hydrogen bridges (Fig. 5), 3 of them are with residues located at the LBD in GluN2B (Thr 514, Ser 690, Tyr 762). The EAR18 Asp 4 residue plays an important role in establishing these 3 hydrogen bridges (Fig. 5). The Tyr 5 of EAR18 contributes to the formation of a hydrophobic interaction (Pi–sigma) with the Ile 534 of the GluN2B, and the Ala 8 of EAR18 is forming and hydrogen bridge with the Lys 485 of the GluN2B. At the LBD, the EAR18 conformation is less extended than that of the EAR16, allowing EAR18 to interact with a larger number of residues in the GluN2B.

## Discussion

In this study we demonstrated that the peptides EAR16 and EAR18 can inhibit NMDA-evoked currents in hippocampal neurons in a dose-dependent and highly reversible manner. We also demonstrated that these peptides inhibited NMDA-evoked currents evoked by recombinant NMDAr containing the GluN1a–GluN2B subunits. However, only EAR16 showed higher selectivity for GluN1a–GluN2B over GluN1a–GluN2A.

Conantokin-G is a 17 amino acid peptide (MW 2264.2 Da) blocks NMDA-evoked currents in hippocampal [45] and cortical [19] neurons. In expression systems, conantokin-G has been shown to be selective for NMDAr containing the GluN2B subunit [19]. EAR16 and EAR18 were designed based on evaluations of in silico interactions by using point mutation on the conantokin-G sequence and by evaluating their binding capacity to an extracellular site of the GluN2B receptor. EAR16 and EAR18 also have 17 amino acids, between them their sequence differs in two amino acids. Both peptides have the same calculated isoelectric point of 3.76, which is higher to that reported for conantokin-G of 2.02 [46]. EAR16 and EAR18 peptides do not have γ-carboxiglutamic acid (Gla) which is present in conantokin-G. The latter was chosen to increase the flexibility of the peptide’s structure, with the rationale that this would facilitate their interactions with the conantokin-G binding pocket in the GluN2B subunit.

**Fig. 6:**
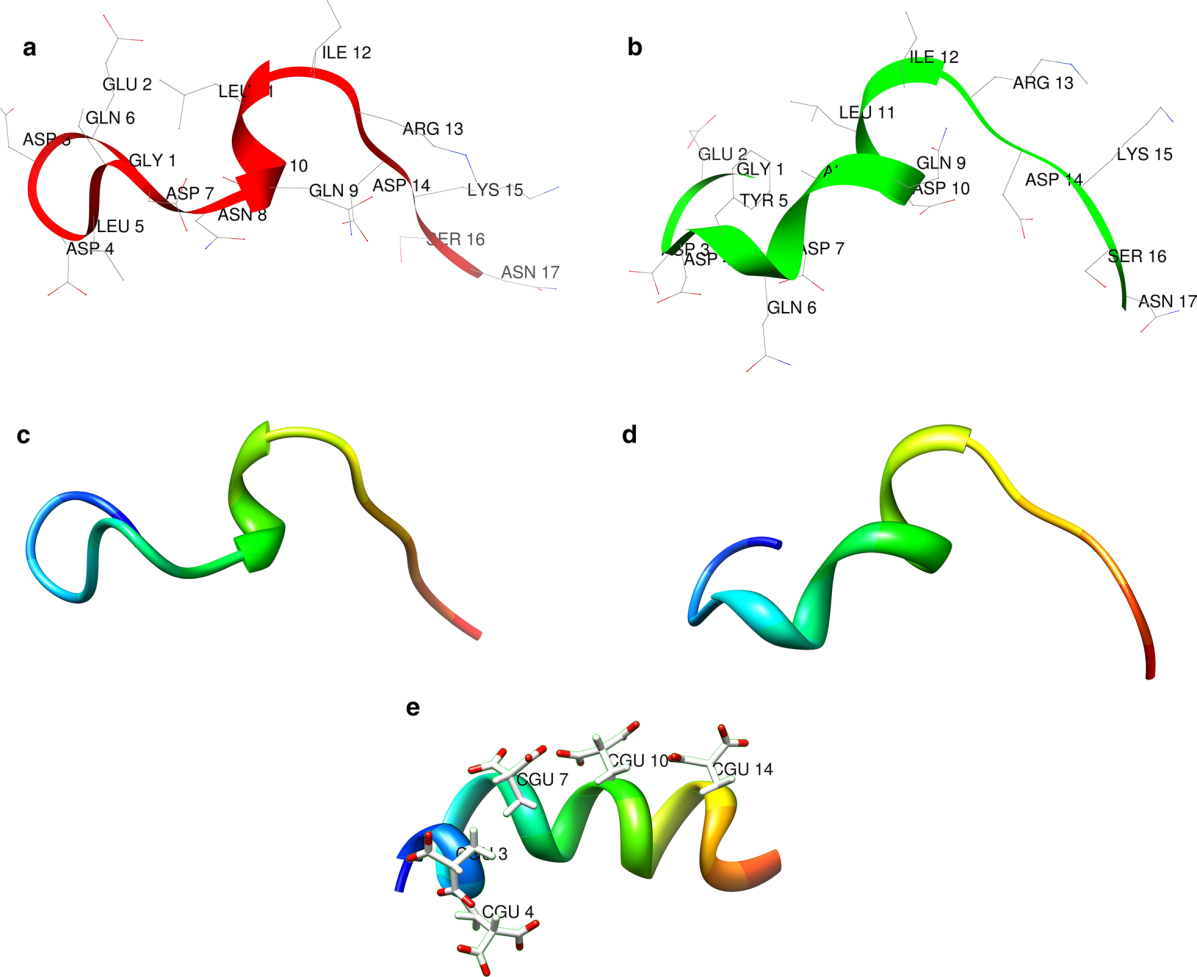
Schematic representation (backbone ribbons) of peptides. The predicted structures for EAR16 (**a**, **c**) and EAR18 (**b**, **d**), and the NMR reported structure for Con-G (**e**) (PDB: 1ONU [48]) are shown. The 3D predicted structure for EAR16 and EAR18 was performed using a hydrophilic environment, both peptides EAR16 and EAR18 shown a non-compact helical conformation compared with Con-G. The N-terminal is represented in blue. The images were rendered using UCSF Chimera software (https://www.cgl.ucsf.edu/chimera/)

The prediction for the three-dimensional structure for EAR18 and EAR16 was performed using a hydrophilic environment. Figure 6 shows the predicted structure for EAR18 and EAR16 and the one reported for conantokin-G [47, 48]. While conantokin-G adopts a helical conformation in more than 70% of its sequence [47, 48], EAR18 and EAR16 adopt a helical conformation in 50% (EAR18) or less (EAR16) of their sequence. The high level of helical conformation in conantokin-G is due to the presence of Gla, which coordinates binding of 4 calcium ions [48–50]. The decrease in the proportion of helical conformation will increase the molecular flexibility of EAR18 and EAR16 and this may contribute to the observed high reversibility of EAR16 and EAR18 (present study). Conantokin-G displays a very slow and incomplete reversibility in cortical neurons, such that after 3 min of washout only about 10% of the NMDA-evoked current was recuperated [19]. In the same study, it was found that the reversibility of conantokin-G was faster and more complete in oocytes expressing GluN1a–GluN2B, such that after 4 min of washout the NMDA-evoked currents displayed about 80% recovery [19]. We observed that EAR16 and EAR18 both were highly reversible in both hippocampal and in HEK cells expressing recombinant NMDAr, but again it also appears that their reversibility was faster from the recombinant NMDAr than from NMDAr expressed in neuronal cells. Together, the results indicate that the conformation of the ligand binding domain (LBD) in the GluN2 subunits may be affected by their neuronal environment. Regardless of such difference we found that EAR16 and EAR18 were highly reversible. This high reversibility represents an advantage for the use of these peptides as potential pharmacological agents in comparison to other available NMDAr blockers, including general NMDAr blockers ((+)MK801) and those selective for GluN2B (conantokin-G, Ro 25-6981).

The Met 739 residue in GluN2B, is not present in the GluN2A subunit and has been reported to be a molecular determinant for the selective antagonist of conantokin-G on GluN2B over the GlunN2A subunit [44, 51]. The Docking results show the formation of a hydrophobic (alkyl type) interaction between the Leu 11 of EAR16 and the Met 739-GluN2B, such interaction is not observed for any of the residues of EAR18. This interaction may contribute to the apparent higher selectivity of EAR16 over EAR18 for the GluN2B subunit over the GluN2A subunit.

Previously by testing various conantokin analogs, it was found that the presence and location of Gla appeared to be important to confer to the conantokin analogs selectivity for the GluN2B subunit over the GluN2A subunit [18]. We found that EAR18, in which Gla was replaced by Asp, did not display selectivity between GluN2B and GluN2A, which is consistent with the idea that Gla is important to confer selectivity for GluN2B. However, we also found that EAR16, in which Gla was also replaced by Asp, showed higher selectivity for GluN2B over GluN2A, indicating that Gla is not the only residue conferring selectivity for GluN2B over GluN2A.

There are only two amino acids difference between EAR18 and EAR16, in EAR16 these are a Leucine (Leu 5) and an asparagine (Asn 8), which in EAR18 correspond to tyrosine (Tyr 5) and alanine (Ala 8). Our results are consistent with previous observation in which it has been reported that replacement of Leu 5 with Tyr in conantokin-G results in a reduction on the conantokin-G selectivity for GluN2B [18, 52].

To determine whether the amino acid substitutions in EAR16 results in selectivity for only the GluN2B, additional studies are required measuring its selectivity for GluN2B over the other two GluN2 subunits, the GluN2C and GLuN2D.

With respect to the predicted free energy of binding, one would expect that EAR18 (−7.7 kcal/mol) would have a higher affinity and potentially a higher interaction with GluN2B than EAR16 (−6.6 kcal/mol). However, their affinity is comparable, and EAR16 shows an apparent higher selectivity for GluN2B than GluN2A, than EAR18. Hence, neither the lower free energy of binding or the higher number of interactions of EAR18 over EAR 16 predict selectivity for GluN2B with respect to GluN2A.

The IC50 value for EAR16 and EAR18 is high as compared to that displayed by conantokin-G. The reported IC50 for conantokin-G is 0.48 µM in hippocampal [45] and cortical [19] neurons, and 0.1 µM in Xenopus oocytes expressing the GluN1a–GluN2B subunits [51]. Hence our results with EAR16 and EAR18 suggest that the Gla residue contributes to the high affinity of conantokin-G.

This study has several limitations. First, we did not measure whether the peptide block was voltage-dependent; neither whether the peptide block was affected by the presence of physiological levels of Mg^2+^. Second, we measured the selectivity of these peptides between GluN2B and GluN2A. This was done in part because the amino acid sequence corresponding to that used for the GluN2B model (1-818 amino acids) is conserved (71.9% identity) and of the LBD (44 amino acids) is highly conserved (95.5% identity) between GluN2A and BluN2B [25, 41, 42]. However, although to a lower extent, the corresponding sequences in the GluN2B subunit also show conservation with the GluN2C (1-818 amino acids, 60.2% identity; LBD 90.9% identity) and with the GluN2D (1-818 amino acids, 57.6% identity, LBD 93.2% identity) subunits [42]. Hence determining the potency of these peptides on recombinant NMDAr containing other GluN2 and GluN3 subunits, would provide additional information about their level of selectivity which will help define their potential use as pharmacological agents.

## Conclusions

We found that EAR16 and EAR18 block in a reversible manner NMDA-evoked currents in hippocampal neurons. Moreover, EAR16 showed selectivity for the GluN2B over GluN2A. The high reversibility of both of these peptides and the selectivity of EAR16 for GluN2B, make them attractive as potential pharmacological agents.

## Abbreviations

NMDA: *N*-methyl-d-aspartate
NMDAr: *N*-methyl-d-aspartate receptor
IC50: half maximal inhibitory concentration
Emax: maximal inhibitory effect
iGluRs: inotropic glutamate receptors
ATD: amino terminal domain
ABD: agonist/ligand binding domain
LBD: ligand binding domain
TMD: trans-membrane domain
CTD: carboxyl terminal domain
M.W.: molecular weight
MS (M + H^+^): hydrogen cation mass spectrometry analysis
MS (M + Na^+^): sodium ion mass spectrometry analysis
UCSF: University of California San Francisco
HPLC: high-performance liquid chromatograph
PEPstr: Peptide Tertiary Structure Prediction Server
AMPA: α-amino-3-hydroxy-5-methyl-4-isoxazolepropionic acid
ANOVA: analysis of variance
Gla: γ-carboxiglutamic acid

## References

[1] Barria A, Malinow R (2005). NMDA receptor subunit composition controls synaptic plasticity by regulating binding to CaMKII. Neuron.

[2] Gardoni F, Polli F, Cattabeni F, Di Luca M (2006). Calcium-calmodulin-dependent protein kinase II phosphorylation modulates PSD-95 binding to NMDA receptors. Eur J Neurosci.

[3] Fan X, Jin WY, Wang YT (2014). The NMDA receptor complex: a multifunctional machine at the glutamatergic synapse. Front Cell Neurosci.

[4] Paoletti P, Neyton J (2007). NMDA receptor subunits: function and pharmacology. Curr Opin Pharmacol.

[5] Carvajal FJ, Mattison HA, Cerpa W (2016). Role of NMDA receptor-mediated Glutamatergic signaling in chronic and acute neuropathologies. Neural Plast.

[6] Laube B, Kuhse J, Betz H (1998). Evidence for a tetrameric structure of recombinant NMDA receptors. J Neurosci.

[7] Matsuda S, Kamiya Y, Yuzaki M (2005). Roles of the N-terminal domain on the function and quaternary structure of the ionotropic glutamate receptor. J Biol Chem.

[8] Sobolevsky AI, Rosconi MP, Gouaux E (2009). X-ray structure, symmetry and mechanism of an AMPA-subtype glutamate receptor. Nature.

[9] Low CM, Wee KS (2010). New insights into the not-so-new NR3 subunits of *N*-methyl-d-aspartate receptor: localization, structure, and function. Mol Pharmacol.

[10] Perez-Otano I, Larsen RS, Wesseling JF (2016). Emerging roles of GluN3-containing NMDA receptors in the CNS. Nat Rev Neurosci.

[11] Paoletti P, Bellone C, Zhou Q (2013). NMDA receptor subunit diversity: impact on receptor properties, synaptic plasticity and disease. Nat Rev Neurosci.

[12] Schumann J, Alexandrovich GA, Biegon A, Yaka R (2008). Inhibition of NR2B phosphorylation restores alterations in NMDA receptor expression and improves functional recovery following traumatic brain injury in mice. J Neurotrauma.

[13] Dingledine R, Borges K, Bowie D, Traynelis SF (1999). The glutamate receptor ion channels. Pharmacol Rev.

[14] Furukawa H, Singh SK, Mancusso R, Gouaux E (2005). Subunit arrangement and function in NMDA receptors. Nature.

[15] Yao Y, Mayer ML (2006). Characterization of a soluble ligand binding domain of the NMDA receptor regulatory subunit NR3A. J Neurosci.

[16] Paoletti P, Ascher P, Neyton J (1997). High-affinity zinc inhibition of NMDA NR1-NR2A receptors. J Neurosci.

[17] Williams K (1993). Ifenprodil discriminates subtypes of the *N*-methyl-d-aspartate receptor: selectivity and mechanisms at recombinant heteromeric receptors. Mol Pharmacol.

[18] Sheng Z, Prorok M, Castellino FJ (2010). Specific determinants of conantokins that dictate their selectivity for the NR2B subunit of *N*-methyl-d-aspartate receptors. Neuroscience.

[19] Donevan SD, McCabe RT (2000). Conantokin G is an NR2B-selective competitive antagonist of *N*-methyl-d-aspartate receptors. Mol Pharmacol.

[20] Trott O, Olson AJ (2010). AutoDock Vina: improving the speed and accuracy of docking with a new scoring function, efficient optimization, and multithreading. J Comput Chem.

[21] London N, Raveh B, Cohen E, Fathi G, Schueler-Furman O (2011). Rosetta FlexPepDock web server-high resolution modeling of peptide-protein interactions. Nucleic acids research.

[22] Reyes-Guzman E, Vega-Castro NA, Reyes-Montano EA. Diseño de péptidos inhibidores de interacciones de la subunidad GluN2B del receptor NMDA en isquemia. Rev Cienc Tecnol (RECyT). 2017;27:ISSN:1851–7587 **(in press)**.

[23] Pettersen EF, Goddard TD, Huang CC, Couch GS, Greenblatt DM, Meng EC, Ferrin TE (2004). UCSF Chimera—a visualization system for exploratory research and analysis. J Comput Chem.

[24] Zhang Y (2008). I-TASSER server for protein 3D structure prediction. BMC Bioinform.

[25] Traynelis SF, Wollmuth LP, McBain CJ, Menniti FS, Vance KM, Ogden KK, Hansen KB, Yuan H, Myers SJ, Dingledine R (2010). Glutamate receptor ion channels: structure, regulation, and function. Pharmacol Rev.

[26] Laskowski RA, Hutchinson EG, Michie AD, Wallace AC, Jones ML, Thornton JM (1997). PDBsum: a Web-based database of summaries and analyses of all PDB structures. Trends Biochem Sci.

[27] Morris AL, MacArthur MW, Hutchinson EG, Thornton JM (1992). Stereochemical quality of protein structure coordinates. Proteins.

[28] Raveh B, London N, Schueler-Furman O (2010). Sub-angstrom modeling of complexes between flexible peptides and globular proteins. Proteins.

[29] Longart M, Liu Y, Karavanova I, Buonanno A (2004). Neuregulin-2 is developmentally regulated and targeted to dendrites of central neurons. J Comp Neurol.

[30] Beaudoin GM, Lee SH, Singh D, Yuan Y, Ng YG, Reichardt LF, Arikkath J (2012). Culturing pyramidal neurons from the early postnatal mouse hippocampus and cortex. Nat Protoc.

[31] Luo JH, Fu ZY, Losi G, Kim BG, Prybylowski K, Vissel B, Vicini S (2002). Functional expression of distinct NMDA channel subunits tagged with green fluorescent protein in hippocampal neurons in culture. Neuropharmacology.

[32] Hamill OP, Marty A, Neher E, Sakmann B, Sigworth FJ (1981). Improved patch-clamp techniques for high-resolution current recording from cells and cell-free membrane patches. Pflugers Arch.

[33] Shelley C (2015). Single-channel recording of glutamate receptors. Curr Protoc Pharmacol.

[34] Huang L, Balsara RD, Sheng Z, Castellino FJ (2010). Conantokins inhibit NMDAR-dependent calcium influx in developing rat hippocampal neurons in primary culture with resulting effects on CREB phosphorylation. Mol Cell Neurosci.

[35] Mayer ML, Westbrook GL, Guthrie PB (1984). Voltage-dependent block by Mg^2+^ of NMDA responses in spinal cord neurones. Nature.

[36] Nowak L, Bregestovski P, Ascher P, Herbet A, Prochiantz A (1984). Magnesium gates glutamate-activated channels in mouse central neurones. Nature.

[37] Loftis JM, Janowsky A (2003). The *N*-methyl-d-aspartate receptor subunit NR2B: localization, functional properties, regulation, and clinical implications. Pharmacol Ther.

[38] Thomas CG, Miller AJ, Westbrook GL (2006). Synaptic and extrasynaptic NMDA receptor NR2 subunits in cultured hippocampal neurons. J Neurophysiol.

[39] Tovar KR, Westbrook GL (2002). Mobile NMDA receptors at hippocampal synapses. Neuron.

[40] Fischer G, Mutel V, Trube G, Malherbe P, Kew JN, Mohacsi E, Heitz MP, Kemp JA (1997). Ro 25-6981, a highly potent and selective blocker of *N*-methyl-d-aspartate receptors containing the NR2B subunit. Characterization in vitro. J Pharmacol Exp Ther.

[41] Wyllie DJ, Livesey MR, Hardingham GE (2013). Influence of GluN2 subunit identity on NMDA receptor function. Neuropharmacology.

[42] Ishii T, Moriyoshi K, Sugihara H, Sakurada K, Kadotani H, Yokoi M, Akazawa C, Shigemoto R, Mizuno N, Masu M (1993). Molecular characterization of the family of the *N*-methyl-d-aspartate receptor subunits. J Biol Chem.

[43] Karakas E, Furukawa H (2014). Crystal structure of a heterotetrameric NMDA receptor ion channel. Science.

[44] Sheng Z, Liang Z, Geiger JH, Prorok M, Castellino FJ (2009). The selectivity of conantokin-G for ion channel inhibition of NR2B subunit-containing NMDA receptors is regulated by amino acid residues in the S2 region of NR2B. Neuropharmacology.

[45] Klein RC, Galdzicki Z, Castellino FJ (1999). Inhibition of NMDA-induced currents by conantokin-G and conantokin-T in cultured embryonic murine hippocampal neurons. Neuropharmacology.

[46] Kaas Q, Yu R, Jin AH, Dutertre S, Craik DJ (2012). ConoServer: updated content, knowledge, and discovery tools in the conopeptide database. Nucleic Acids Res.

[47] Cnudde SE, Prorok M, Dai Q, Castellino FJ, Geiger JH (2007). The crystal structures of the calcium-bound con-G and con-T[K7γ] dimeric peptides demonstrate a metal-dependent helix-forming motif. J Am Chem Soc.

[48] Skjaerbaek N, Nielsen KJ, Lewis RJ, Alewood P, Craik DJ (1997). Determination of the solution structures of conantokin-G and conantokin-T by CD and NMR spectroscopy. J Biol Chem.

[49] Chandler P, Pennington M, Maccecchini ML, Nashed NT, Skolnick P (1993). Polyamine-like actions of peptides derived from conantokin-G, an *N*-methyl-d-aspartate (NMDA) antagonist. J Biol Chem.

[50] Lin SY, Wu K, Len GW, Xu JL, Levine ES, Suen PC, Mount HT, Black IB (1999). Brain-derived neurotrophic factor enhances association of protein tyrosine phosphatase PTP1D with the NMDA receptor subunit NR2B in the cortical postsynaptic density. Brain Res Mol Brain Res.

[51] Teichert RW, Jimenez EC, Twede V, Watkins M, Hollmann M, Bulaj G, Olivera BM (2007). Novel conantokins from Conus parius venom are specific antagonists of *N*-methyl-d-aspartate receptors. J Biol Chem.

[52] Klein RC, Prorok M, Galdzicki Z, Castellino FJ (2001). The amino acid residue at sequence position 5 in the conantokin peptides partially governs subunit-selective antagonism of recombinant *N*-methyl-d-aspartate receptors. J Biol Chem.

